# Safety and Efficacy of Rapamycin-Eluting Vertebral Stents in Patients With Symptomatic Extracranial Vertebral Artery Stenosis

**DOI:** 10.3389/fneur.2021.649426

**Published:** 2021-11-26

**Authors:** Gaoting Ma, Ligang Song, Ning Ma, Raynald, Jie Shuai, Wei Wu, Jieqing Wan, Zhenwei Zhao, Guangjian Li, Sen Yin, Shenghao Ding, Jiang Li, Baixue Jia, Xu Tong, Dapeng Mo, Feng Gao, Xuan Sun, Yiming Deng, Xiaochuan Huo, Wei Li, Kangning Chen, Zhongrong Miao

**Affiliations:** ^1^Department of Interventional Neuroradiology, Beijing Tiantan Hospital, Capital Medical University, Beijing, China; ^2^Department of Neurology, Xinqiao Hospital, Army Medical University, Chongqing, China; ^3^Department of Neurology, Qilu Hospital, Shandong University, Jinan, China; ^4^Department of Neurosurgery, Renji Hospital, Shanghai Jiaotong University School of Medicine, Shanghai, China; ^5^Department of Neurosurgery, Institute for Functional Neurosurgery P.L.A, TangDu Hospital, Fourth Military Medical University, Xi'an, China; ^6^Department of Neurology, Southwest Hospital, Third Military Medical University, Chongqing, China; ^7^State Key Laboratory of Cardiovascular Disease, Fuwai Hospital, National Center for Cardiovascular Diseases, Chinese Academy of Medical Sciences and Peking Union Medical College, Beijing, China

**Keywords:** vertebral artery stenosis, drug-eluting stent, symptomatic stenosis, in-stent restenosis, objective performance criterion

## Abstract

**Background and Purpose:** Drug-eluting stents generally have superior performance to bare metal stents in the treatment of vertebral artery stenosis (VAS). This prospective, multicenter, and single-arm clinical trial was initiated to assess in-stent restenosis (ISR) and midterm outcome after rapamycin-eluting stent placement in patients with symptomatic extracranial VAS.

**Methods:** The subjects underwent angiographic follow-up at 6 months and final clinical follow-up at 12 months. The primary efficacy endpoint was ISR at 6 months. Secondary endpoints included technical success, target lesion-related transient ischemic attack (TIA), stroke, or death, and all-cause TIA, stroke, or death during the 12-month follow-up period.

**Results:** A total of 104 stents were implanted in the 101 patients and 83 patients (82.2%) completed angiographic follow-up at 6 months. The technical success rate was 86.1% (87/101); mean in-stent stenosis rate was 25.1 ± 17.1% and ISR rate was 5.9% (95% CI: 0.8–10.9%). All the patients with ISR were completely asymptomatic and no stent fractures were observed during angiographic follow-up. At the 12-month clinical follow-up, target lesion-related TIA, stroke, or death had occurred in two (2.0%) patients and all-cause TIA, stroke, or death had occurred in six (6.1%) patients.

**Conclusion:** The placement of rapamycin-eluting stents in patients with symptomatic extracranial VAS yields favorable ISR results and showed a trend of favorable safety outcomes including low rates of perioperative complications and late stroke. However, further study is needed to establish the long-term clinical benefits of this stent in the treatment of VA disease.

## Introduction

Posterior circulation strokes are associated with high morbidity and mortality rates and account for approximately 20% of all the ischemic strokes, with up to 20% of cases involving vertebral artery stenosis (VAS) (1). In patients who are refractory to medical treatment, endovascular treatment by balloon angioplasty or stenting is recommended (2–6). However, endovascular stenting was shown to be superior to balloon angioplasty, as it yields immediate results and has a low rate of periprocedural complications (7).

Despite the promising results achieved with endovascular stenting, high rates of in-stent restenosis (ISR) ranging from 11.1 to 66.7% have been reported (5, 8–10), which is mainly caused by neointimal hyperplasia. To overcome this problem, drug-eluting stents (DESs) were developed for the treatment of severe coronary artery stenosis. Both the paclitaxel and rapamycin are the commonly used drugs for DES, with the latter shown to be more effective for preventing coronary ISR (11, 12). Additionally, DESs have shown promising results in the treatment of cerebrovascular stenosis. However, most of these studies were case reports or case series and there are limited comparative data on the efficacy of rapamycin- and paclitaxel-eluting stents in the treatment of VAS. The former is increasingly being used because of its low neurotoxicity, but its safety has yet to be validated in a large sample.

It is worth noting that DESs used to treat VAS in previous studies were “off-label” and only indicated for coronary artery stenosis. A prospective, multicenter, and single-arm safety and efficacy evaluation of a rapamycin-eluting stent specifically indicated for VAS was recently completed. In this study, we investigated the applicability of rapamycin-eluting stents to the treatment of symptomatic extracranial VAS.

## Materials and Methods

### Study Design and Population

This prospective, single-arm clinical trial based on objective performance criteria was carried out at six high-volume centers. Eligible patients were between 18 and 80 years of age and presented with symptomatic extracranial VAS resulting from presumed arteriosclerotic disease, defined as posterior circulation stroke or transient ischemic attack (TIA) in the previous 90 days despite receiving intensive antiplatelet therapy (with aspirin and clopidogrel) and management of risk factors (13, 14). Angiographic inclusion criteria were lesion length ≤ 21 mm and degree of stenosis ≥ 50% [i.e., the Warfarin-Aspirin Symptomatic Intracranial Disease (WASID) trial definition] (15–18).

Key clinical exclusion criteria were tandem stenoses and previous surgical or endovascular intervention in the target lesion area; a potential cause of stroke or TIA other than stenosis in a VA (e.g., atrial fibrillation or lacunar stroke); severe neurologic dysfunction (the Modified Rankin Scale score ≥3); myocardial infarction within 2 weeks of the procedure; excessive tortuosity or severe calcification of the target lesion; non-atherosclerotic lesion; other concurrent intracranial diseases such as intracranial hemorrhage, aneurysm, arteriovenous malformation, and/or intracranial tumor; severe renal dysfunction; and allergy or other contraindications to oral antiplatelet medication, rapamycin and its derivatives, cobalt-base alloy, polylactic acid, or steel.

The protocol was approved by the Institutional Review Board or Ethics Committee of each participating hospital. A written informed consent was obtained from each patient prior to enrollment. The trial was conducted in compliance with Chinese medical device regulations.

### Device Description

**Figure 1:**
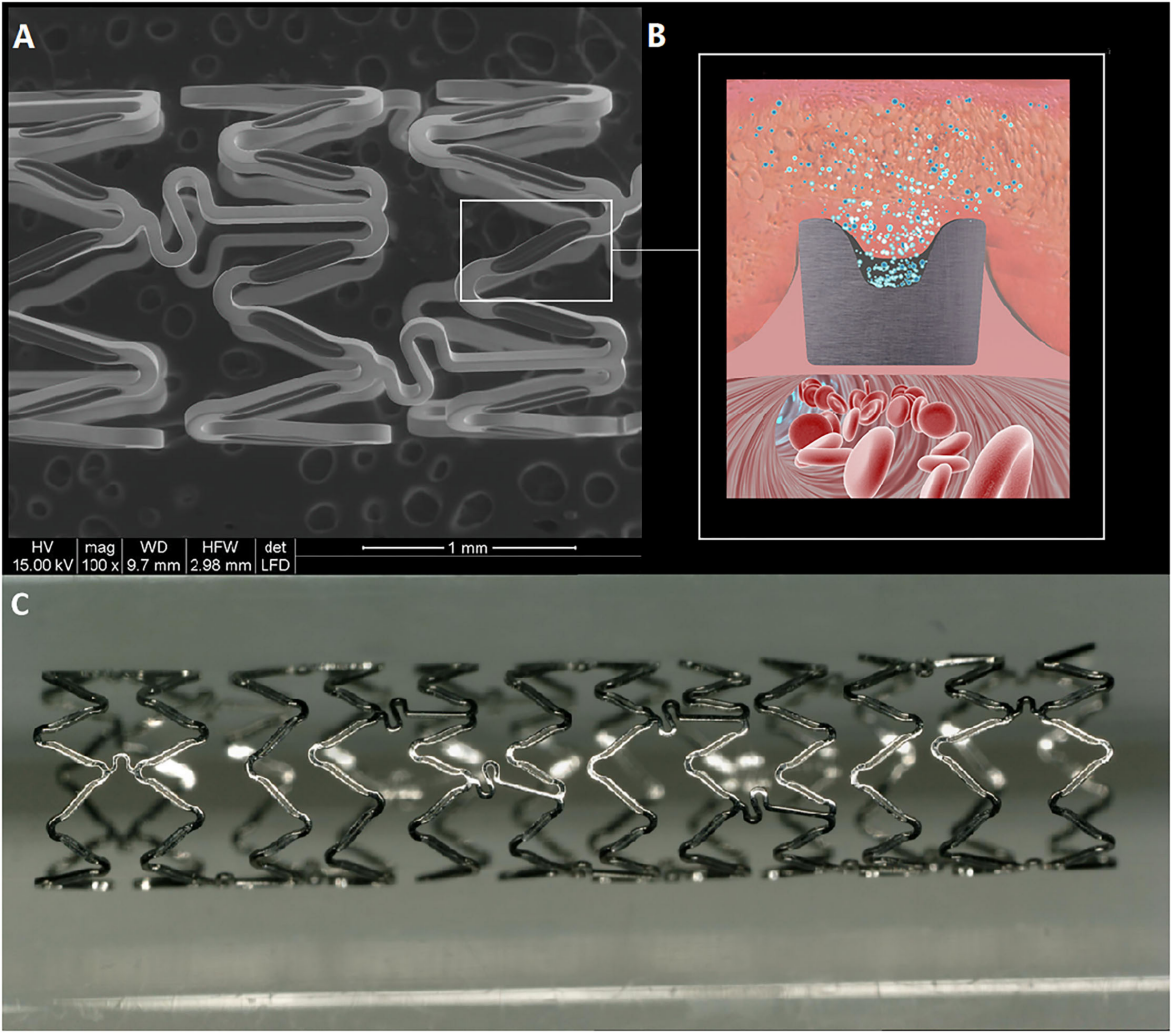
The Firehorus stent design. **(A)** The Firehorus stent is made of L605 cobalt chromium alloy; **(B)** Abluminal surface with recessed grooves containing D,L-polylactic acid biodegradable polymer, which provides controlled rapamycin (sirolimus) release; and **(C)** The Firehorus stent.

The Firehorus Rapamycin Target-eluting Vertebral Artery Stent System (Shanghai Microport NeruoTech, Shanghai, China) is a novel balloon-expandable stent fabricated from L605 cobalt chromium alloy with a strut thickness of 86 μm (Figure 1). Recessed grooves on the abluminal surface contain a D,L-polylactic acid biodegradable polymer of 10 μm thickness, which provides controlled release of the antiproliferative drug rapamycin. The remaining three sides of the stent strut are devoid of drug or polymer. The rapamycin density is 0.3 μg/mm^2^, with approximately 90% released by 90 days postimplantation. The stent is premounted on a custom rapid-exchange balloon delivery catheter system to avoid injury or distortion of the coating during the crimping process. The Firehorus stents were available for this trial in diameters of 2.25–4.0 mm and lengths of 13–23 mm.

### Procedure

Dual antiplatelet therapy consisting of 100 mg aspirin plus 75 mg clopidogrel once daily was administered at least 3 days prior to the procedure. A loading dose of 300 mg aspirin plus 300 mg clopidogrel was given, if the procedure was scheduled to begin immediately. All the patients were evaluated for aspirin and clopidogrel resistance with the VerifyNow Platelet Function Assay (Accumetrics, San Diego, California, USA). Stent implantation was carried out according to the instructions of the manufacturer provided with each device and current hospital and neurovascular standard practices. All the procedures were routinely performed under local anesthesia without intravenous sedation. Procedures were performed via the transfemoral or transradial route, which was selected based on the most stable guiding catheter position for treatment. Heparin was administered to maintain an activated clotting time of 250–300 s. A 6F guiding catheter was placed into the subclavian artery proximal to the origin of the target VA. At this point, a 0.014-inch guidewire was advanced across the lesions. In a minority of cases, pre-dilation with a coronary balloon catheter was performed in order to facilitate the passage of the rapamycin-eluting stent, which was deployed across the stenosis. Post-dilation was not routinely performed. At the end of the procedure, an angiogram was performed to measure residual stenosis. The combined antiplatelet medication (100–300 mg aspirin and 75 mg clopidogrel daily) was continued for at least 1 year postimplantation. Intensive management of risk factors after stent implantation was continued in all the patients (13, 14). Protocol-specified angiographic follow-up was required at 6 months (±30 days) posttreatment. Clinical follow-up was scheduled at 1, 6, and 12 months postimplantation.

### Endpoints and Definitions

The primary endpoint was ISR at 6 months (±30 days), defined as ≥50% stenosis within the stent or just outside the stent margins (19). Technical success was defined as residual stenosis of ≤20% after final treatment with the DES. Safety endpoints included target lesion-related TIA, stroke, or death and all-cause TIA, stroke, or death during the follow-up period. Stroke was defined as a focal neurologic deficit lasting more than 24 h. Target lesion-related stroke was defined as clinical features indicative of stroke of the brainstem, cerebellum, or occipital lobe. Posterior circulation TIA was defined as a transient episode of neurologic dysfunction caused by posterior circulation ischemia without acute infarction (20). If a new stroke was suspected, a CT or MRI scan was performed for verification. All the serious adverse events and safety endpoints were adjudicated by a Clinical Endpoint Committee. All the angiographic endpoints were evaluated by an independent core laboratory.

### Statistical Methods

#### Performance Goal and Sample Size

The performance goal in this present study was determined based on a systematic review of 27 articles reporting ISR rates (21); the mean ISR rate was 11% in patients with the DES and 30% in patients treated with a bare metal stent (BMS). It was evident that compared to BMS, DES offered a mean net benefit of 19% for ISR (30–11%). Since the Firehorus is a new DES to treat vertebral stenosis, an absolute difference of 9.5% (half of the benefit) was used to calculate a performance goal of 20.5% (11% plus the prespecified margin of 9.5%). Allowing for a 20% loss to follow-up for the primary endpoint, a sample size of 100 patients was deemed necessary for 80% power to reject the null hypothesis, with a two-tailed **α**-value of 0.05.

#### Statistical Analysis

The primary analysis was based on the intention-to-treat (ITT) principle, defined as enrollment in the study and attempted placement of the rapamycin-eluting stent. Demographics, lesion characteristics, procedural characteristics, and outcome variables of the patient were analyzed with descriptive statistics. Continuous variables are reported as mean ± SD, median with interquartile range, and maximum and minimum values. Categorical variables are reported as counts and percentages. The Kaplan–Meier analysis was used to assess the primary endpoint and determine 95% CI. Sensitivity analysis was performed using the tipping point method to estimate the rate of ISR and 95% CI at 6 months after the procedure. All the statistical analyses were performed using SAS software version 9.4 (SAS Institute, Cary, North Carolina, USA).

## Results

### Trial Population With Baseline and Procedural Characteristics

**Figure 2:**
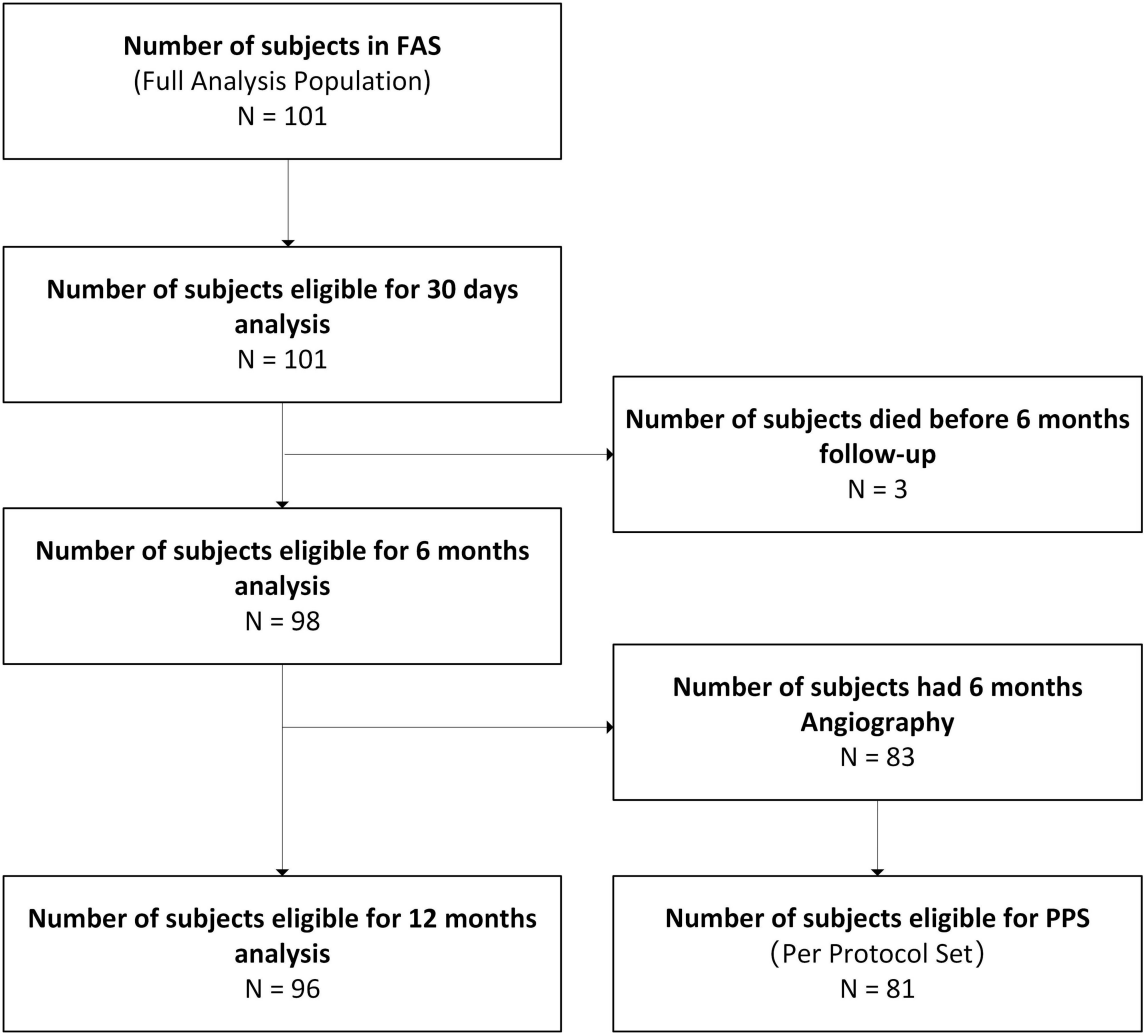
Flowchart of patient selection.

Between July 7, 2014, and November 26, 2015, a total of 101 patients were enrolled in the trial and 104 stents [102 DESs and 2 BMSs (Apollo, MicroPort Scientific, Shanghai, China)] were implanted (1.03 stents per patient) (Figure 2). Baseline demographics, clinical conditions, angiographic characteristics, and procedural data for the patients are shown in Table 1. The mean age of the subjects was 62.87 ± 8.41 years and 18.8% were female; 57 (56.4%) patients had stroke, 37 (36.6%) patients had TIA, and 7 (6.9%) patients had stroke combined with TIA. Cerebrovascular risk factors were highly prevalent including hypertension in 78.2% patients, current or previous smoking in 49.5% patients, hyperlipidemia in 41.6% patients, and diabetes in 28.7% patients. Complete baseline and postprocedure angiographic data were available for the 104 treated lesions. Cerebral angiography showed that all the patients suffered from stenosis in the dominant VA, of which 24 (23.8%) patients suffered from contralateral VA hypoplasia or occlusion. The target lesion involved the V0 segment in 85.6% of patients, V1 segment in 10.6% of patients, V2 segment in 1.9% of patients, and V4 segment in 1.9% of patients. The mean reference vessel diameter was 3.49 ± 0.63 mm and mean lesion diameter was 1.16 ± 0.39 mm.

**Table 1:** Baseline characteristics and pre/post-procedure angiographic results.

**Characteristic**	**Patients (*n* = 101)**
Age, years	62.87 ± 8.41
Female	19 (18.8)
History of diabetes	29 (28.7)
Insulin therapy	12 (41.4)
History of hypertension	79 (78.2)
History of hyperlipidemia	42 (41.6)
Current smoking	31 (30.7)
Prior myocardial infraction	4 (4.0)
Prior transient ischaemic attack	44 (43.6)
Prior Ischemic Stroke	64 (63.4)
Target vessel (*n* = 104 lesions)	
V0	90 (86.5)
V1	10(9.6)
V2	2 (1.9)
V3	0 (0.0)
V4	2 (1.9)
Reference vessel diameter, mm	3.49 ± 0.63
Lesion length, mm	7.18 ± 3.35
Minimum luminal diameter, mm	1.16 ± 0.39
Percentage diameter stenosis	66.9 ± 9.5
Total stent length, mm	15.15 ± 2.75
Stent diameter, mm	3.66 ± 0.50
Pre-dilation	8 (7.7)
Post-dilation	16 (15.4)
Final in-stent minimum luminal diameter, mm	3.14 ± 0.53
Final in-stent percentage diameter stenosis	10.59 ± 9.89
Technical success (Lesion level)	90 (86.5)
Technical success (Patient level)	87 (86.1)
Adverse events in the procedure	0(0.0)

*Values are mean ± SD or n (%); Technical success = residual percentage diameter stenosis ≤20%; Percentage diameter stenosis = (1–[D_Stenosis_/D_Distal_]) × 100%; Angiographic results analyzed on Core-lab data (except Target vessel, Total stent length, Stent diameter, Pre-dilation, Post-dilation)*.

**Figure 3:**
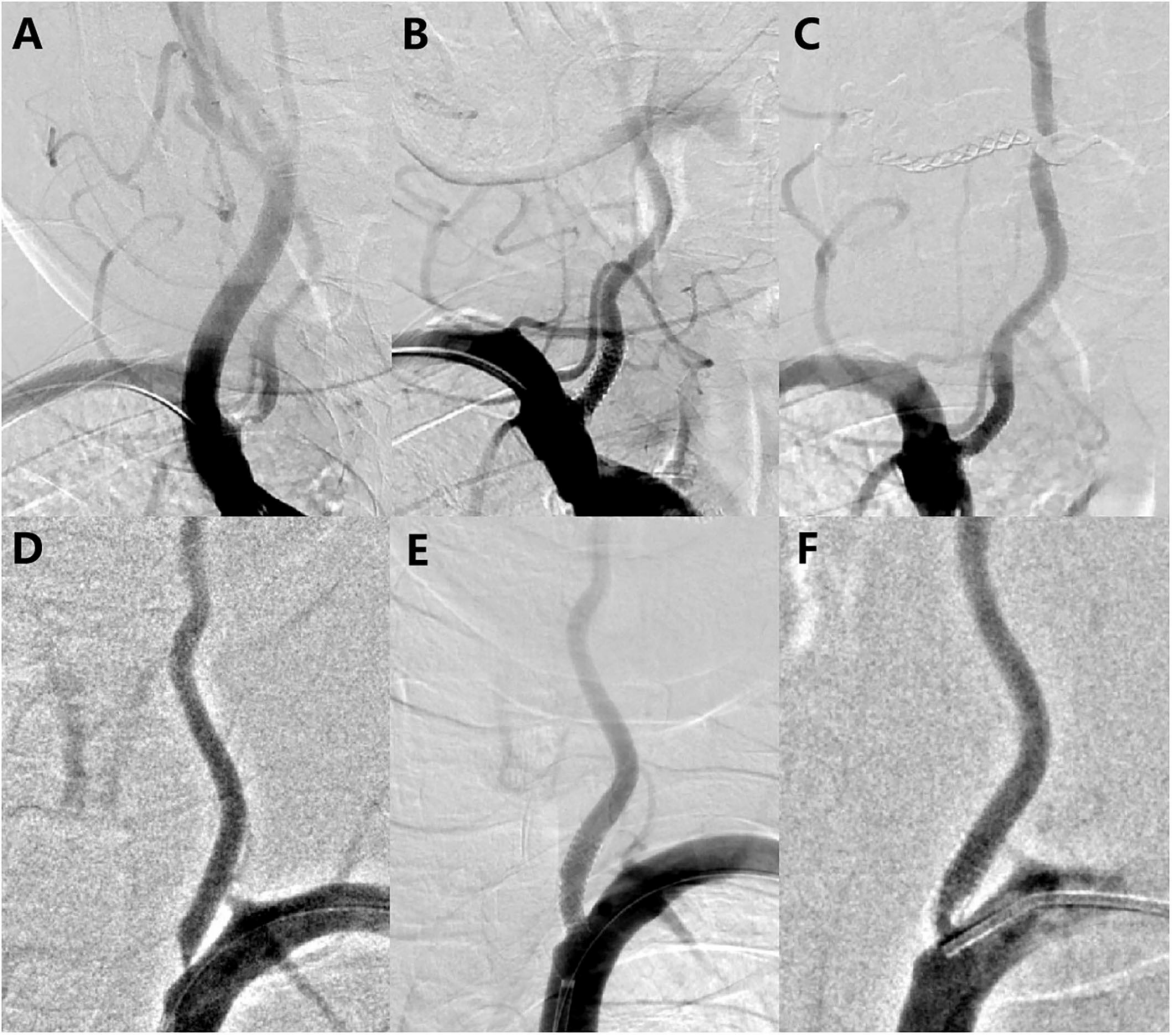
Before and after treatment with the rapamycin-eluting stent. **(A)** Left vertebral artery high-grade stenosis before placing a long 16 × 4.0 mm stent; **(B)** Immediately after implantation, demonstrating a wide-open arterial lumen with 4.5% residual stenosis; **(C)** 6-month follow-up angiogram showing approximately 12.7% restenosis; **(D)** Left vertebral artery high-grade stenosis before placing a long 16 × 4.0 mm stent; **(E)** Immediately after implantation, demonstrating a wide-open arterial lumen with 12% residual stenosis; and **(F)** 6-month follow-up angiogram showing approximately 57% restenosis.

The lesions presented an average pretreatment degree of stenosis of 66.86 ± 9.47% and mean lesion length of 7.18 ± 3.35 mm. Predilation was performed in 8 lesions, whereas postdilation was performed in 16 lesions. Technical success was achieved in 86.1% of patients. There were no intraoperative complications and no fatal or non-fatal stroke, in-hospital death, acute or subacute stent thrombosis, or target lesion revascularization occurred during the perioperative period. After stent implantation, the mean percent diameter stenosis was reduced to 10.59 ± 9.89%. Figure 3 shows the digital subtraction angiograms of two subjects treated with the DES for V0 stenosis.

### Angiographic Outcomes at 6 Months

Three subjects (3.0%) died within 6 months and 15 patients (14.8%) declined participation in the invasive follow-up; thus, 83 subjects (82.2%) were assessed for the primary endpoint at 6 months. Two subjects with tandem stenoses (V0 and V4 segments) were excluded from the per protocol set (PPS) and two patients with V2 segment stenosis refused to undergo angiographic follow-up; thus, the PPS comprised only patients with V0 or V1 segment stenosis. In the full analysis set and the PPS, the mean in-stent stenosis rates were 25.1 ± 17.1% and 24.4 ± 16.1%, respectively, and the primary endpoint of 6-month ISR rate was 5/83 (5.9%) and 3/81 (3.7%), respectively (Table 2). A 51-year-old female experienced asymptomatic in-stent occlusion. Baseline angiography showed 77.3% stenosis in the ostium of the right VA. A 3.5 × 13 mm Firehorus stent was implanted without pre- or postdilation. Residual stenosis immediately after stenting was 5.8%. A branch of the right thyrocervical trunk supplied a retrocorporeal artery collateral to the right VA at the 6-month angiographic follow-up. All the ISR subjects were completely asymptomatic and no stent fractures were observed during angiographic follow-up.

**Table 2:** 6-month angiographic results.

**Parameters**	**FAS**	**PPS**
	**101 Subjects/104 Lesions**	**81 Subjects/82 Lesions**
Reference vessel diameter, mm	3.50 ± 0.56	3.50 ± 0.57
Minimum luminal diameter, mm	2.63 ± 0.69	2.65 ± 0.66
Percentage diameter stenosis	25.1 ± 17.1	24.4 ± 16.1
In-stent stenosis (Lesion level)	5 (5.9)	3 (3.7)
In-stent stenosis (Patient level)	5 (6.0)	3 (3.7)

*Values are mean ± SD or n (%); FAS, Full Analysis Set; PPS, Per Protocol Set; In-stent stenosis = percentage diameter stenosis ≥ 50%; Percentage diameter stenosis = (1–[D_Stenosis_/D_Distal_]) × 100%; 6-month angiographic results analyzed on Core-lab data*.

The upper 95% CI of the primary endpoint calculated with the Clopper–Pearson exact method was 10.9%, well below the performance goal of 20.5%. Sensitivity analysis with the tipping point method showed that only if ≥ 50% of patients (i.e., ≥9 patients) had restenosis can reach the threshold required to accept the null hypothesis. However, in the PPS, only 3.7% of patients (3/81) had restenosis at 6 months. There were no differences in baseline characteristics between subjects who were lost to follow-up and those who were not lost to follow-up; accordingly, there was a low probability of a restenosis rate ≥ 50% among the 18 patients for whom there were no 6-month angiographic results. Thus, the DES was associated with a low ISR rate as predicted.

**Figure 4:**
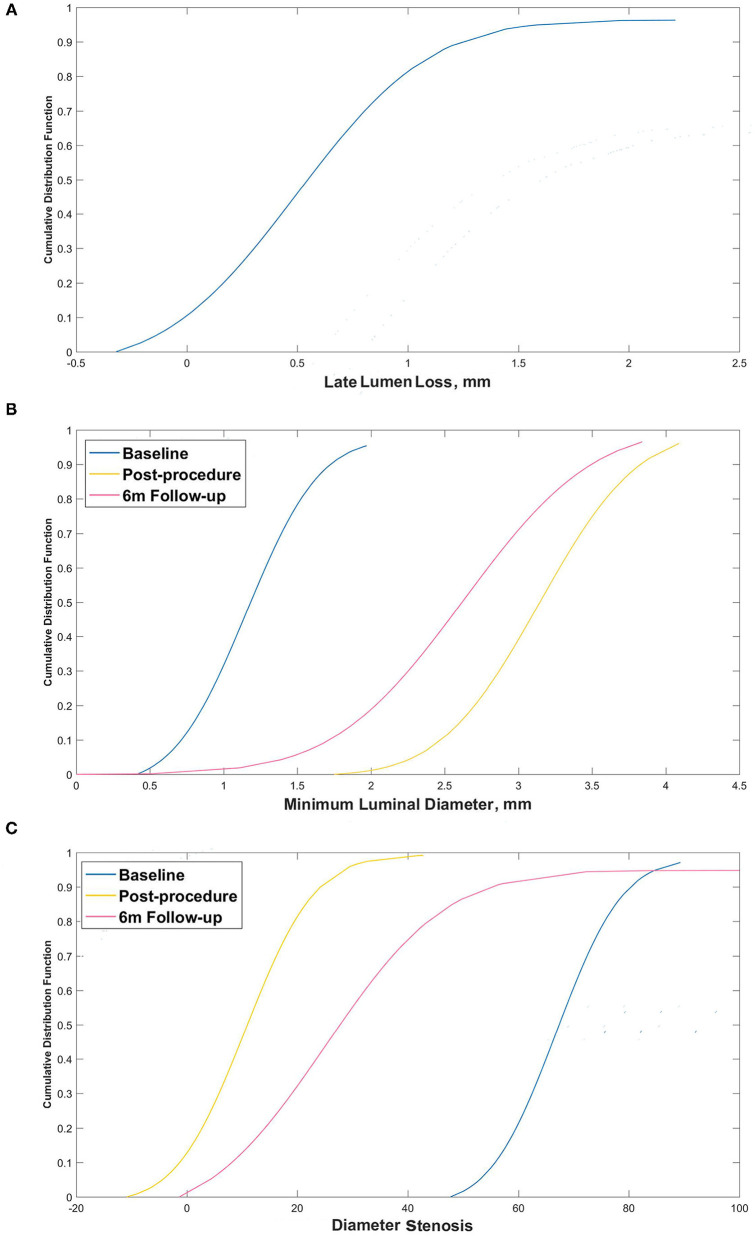
Cumulative frequency distribution curves. **(A)** Cumulative frequency distribution curve for late lumen loss at 6 months; **(B)** Minimum luminal diameter pre- and postprocedure and at the 6-month follow-up; and **(C)** Percentage diameter stenosis pre- and postprocedure and at the 6-month follow-up.

The cumulative distribution frequency for late in-stent lumen loss (LL) is shown in Figure 4A. More than 89% of subjects had LL < 1.0 mm and only one subject had LL > 2.0 mm. The cumulative frequencies of luminal diameter and ISR pre- and postprocedure and at the 6-month follow-up are shown in Figures 4B,C.

### Clinical Outcomes at 12 Months

Clinical follow-up data at 12 months were available for 99 patients (98.0%). Target lesion-related TIA, stroke, or death occurred in two (2.0%) patients including one (1.0%) patient with death and one (1.0%) patient with thalamic hemorrhage; both the events occurred within 6 months. Any TIA, stroke, or death occurred in six (6.1%) patients including three (3.0%) patients with death, one (1%) patient with transient ischemic stroke in the anterior circulation, one (1.0%) patient with anterior circulation ischemic stroke, and one (1.0%) patient with thalamic hemorrhage. Of the three patients who died in the follow-up period, one patient died of ischemic stroke recurrence in the area of the target vessel, one patient died of traumatic cerebral hemorrhage, and one patient died of intestinal tumors. Until the 12-month follow-up, there were 34 serious adverse events in 25 patients, but none was related to either the device or the procedure (Supplementary Table S1).

## Discussion

**Figure 5:**
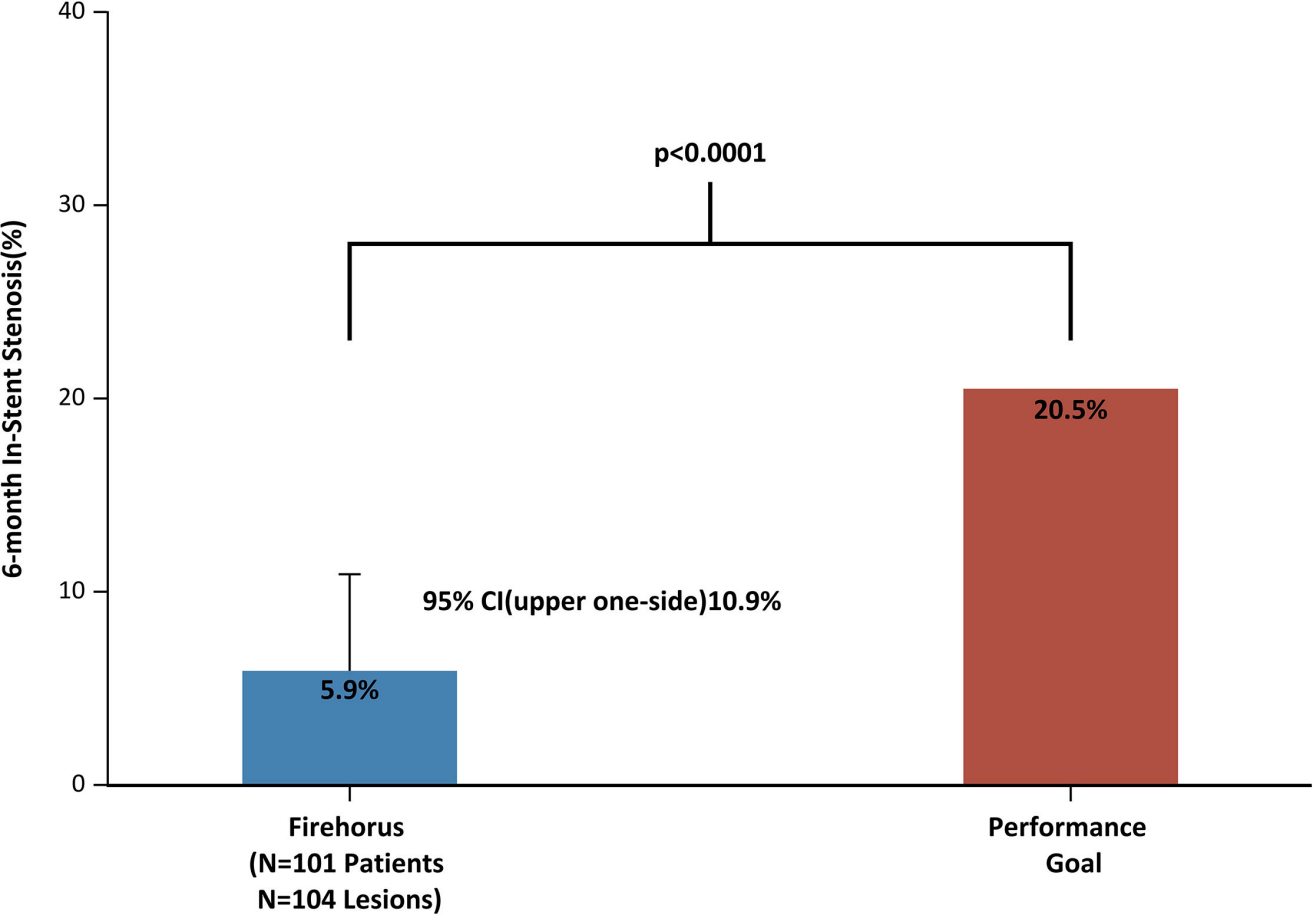
Prespecified performance goal and primary endpoint.

In this prospective, multicenter, and single-arm clinical trial, the rapamycin-eluting stent met the prespecified performance goal for the primary endpoint (Figure 5), supporting the safety and efficacy of the stent for the treatment of symptomatic extracranial VAS. The angiographic endpoints evaluated by the core laboratory showed that the 6-month ISR for the PPS subjects was just 3.7%. This is comparable to the rates reported in other trials of patients treated with a DES in the VA (Table 3) (22–38).

**Table 3:** Summary of reports of vertebral artery angioplasty and stenting with drug-eluting stents.

**Trail**	**Patients (n)**	**Drug-eluting**	**Location**	**Technical success definition**	**Technical success rate**	**Peri-procedural TIA/Stroke**	**Mean imaging follow-up (m)**	**Imaging mode**	**Restenosis definition**	**Restenosis rate**
Gupta et al. (22)	31	PaclitaxelRapamycin	EVA	Successful stent deployment	100%	0	4	DSACTA	>50% Stenosis	7.4% (2/27)
Vajda et al. (23)	48	Paclitaxel	VAO	NR	100%	0	7.7	DSA	>50% Stenosis	12.5% (6/48)
Yu et al. (24)	10	Paclitaxel	VAO	Successful stent deployment	100%	0	12	DSA	>50% Stenosis	0% (0/10)
Ogilvy et al. (25)	15	PaclitaxelRapamycin	VAO	Successful stent deployment	100%	0	NR	CTA	>50% Stenosis	16.7% (2/12)
Park et al. (26)	20	Paclitaxel	VAO	NR	100%	0	14.7	DSA	>50% Stenosis	21.1% (4/19)
Werner et al. (27)	28	Paclitaxel	VAO	Residual stenosis of <20%	100%	0	16	DSA	>50% Stenosis	21.4% (6/28)
Chen et al. (28)	47	PaclitaxelRapamycin	VAO	Residual stenosis of <20%	100%	0	16.3	DSA	>50% Stenosis	5.3% (2/38)
Fields et al. (29)	14	NR	VAO	Successful stent deployment	100%	0	8	DSA	>50% Stenosis	21.4% (3/14)
Song et al. (30)	112	PaclitaxelRapamycin	VA	Residual stenosis of <30%	98.3%	2.7%	43	DSA^*^	>70% Stenosis	6.3% (7/112)
Langwieser et al. (31)	16	Paclitaxel	EVA	Residual stenosis of ≤30%	100%	0	18	DUS	≥70% Stenosis	0% (0/16)
Lu et al. (32)	24	PaclitaxelRapamycin	VAO	Residual stenosis of ≤30%	100%	0	35	DSA	>50% Stenosis	10.0% (2/20)
Raghuram et al. (33)	13	NR	EVA	Successful stent deployment	100%	0	12	DSA	>50% Stenosis	23.1% (3/13)
Che et al. (34)	147	Paclitaxel	VAO	Residual stenosis of <30%	100%	NR	34.8	CTAMRADSA	≥50% Stenosis	8.2% (12/147)
He et al. (35)	20	Rapamycin	VA	Successful stent deployment	100%	0	6.5	DSA	>50% Stenosis or luminal loss >30%	5.0% (1/20)
Maciejewski et al. (36)	148	PaclitaxelRapamycinEverolimusBioliusZotarolimus	EVA	Residual stenosis of <30%	96.7%	1.4%	>6	DUSCTADSA	≥50% Stenosis	27.9% (31/111)
Ortega-Gutierrez et al. (37)	30	ZotarolimusEverolimus	VAO	Successful stent deployment	100%	0	8.8	CTADSA	≥70% Stenosis	7.7% (2/26)
Li et al. (38)	76	NR	VAO	Residual stenosis of <30%	100%	0	12.3	DSACTADUS	>50% Stenosis	18.4% (14/76)

*CTA, computed tomography angiography; DSA, digital subtraction angiography; DUS, duplex ultrasonography; EVA, extracranial vertebral artery; IVA, intracranial vertebral artery; MRA, magnetic resonance angiography; NR, not reported; VA, vertebral artery; VAO, vertebral artery origin*.

* *Patients with recurrent symptoms underwent DSA*.

The efficacy of interventional therapy for symptomatic VAS is controversial. The Carotid and Vertebral Artery Transluminal Angioplasty Study (CAVATAS) failed to show any benefit of VAS intervention (39). However, the result was underpowered due to the small number of patients enrolled in this study. The Vertebral Artery Stenting Trial (VAST) was halted after enrolling 115 patients because of regulatory problems and lack of funding (16). At present, there is no evidence to justify the contraindication of endovascular treatment in patients with medically refractory VAS. Moreover, there are no definite evidence-based guidelines with respect to the role of medical treatments such as risk factor modification and antiplatelet treatment vs. percutaneous transluminal angioplasty and stenting (PTAS) (8, 16, 39). On the other hand, there is no valid reason to withhold PTAS and there is increasing evidence from case series and cohort studies that it is safe and effective, especially at the VA origin (7, 21, 31, 40). The results of this study provide evidence for the safety and efficacy of the rapamycin-eluting stent for the treatment of VAS; ISR rate of 3.7% in the PPS at the 6-month angiographic follow-up was comparable to those reported in recent DES studies (34, 40).

The rapamycin-eluting stent had an excellent safety profile. During the entire follow-up period, target vessel stroke or death occurred in 2.0% (2/99) of subjects and any stroke or death occurred in 6.1% (6/99) of subjects, in contrast to the Stenting of Symptomatic Atherosclerotic Lesions in the Vertebral or Intracranial Arteries (SSYLVIA) study in which the composite 1-year stroke rate associated with target vessels was 13.1% (8/61) (8). Similarly, in the VAST trial, 9% (5/57) of patients in the stenting group had a stroke in the territory of the symptomatic VA during the follow-up period of 1 year.

Despite the low ISR rate and excellent safety profile of the rapamycin-eluting stent in this study, the technical success rate was lower than that reported in other trials with more restricted populations (40). This may be an inherent limitation of an open-label device evaluation where variable behavior according to operator experience cannot be ruled out. In studies with new devices that have a unique mode and method of deployment, there may be a learning curve influence on early applications. Given the broad patient inclusion criteria, unmatched and limited stent sizes (2.25–4.0 mm) may have influenced the rate of technical success. Our result was also related to the more stringent criteria adopted by the independent core laboratory because the technical success rate determined by researchers was as high as 98%. Nonetheless, the difference in technical success did not appear to influence the ISR rate or translate into any differences in safety or efficacy in the ITT population and the PPS in this study.

The antiproliferative activity of rapamycin may contribute to reducing ISR rates by interfering with smooth muscle cell migration and delaying endothelialization; additionally, rapamycin may delay stent thrombosis (41, 42). The coronary DES made with first-generation durable polymer was found to be associated with higher rates of late and very late stent thrombosis, which were partly attributed to hypersensitivity reactions to the polymer (43–45). Antiplatelet therapy is thought to play an important role in reducing the risk of stent thrombosis (46). In the Randomized Study with the Sirolimus-eluting Velocity Balloon-expandable Stent (RAVEL) trial, the rates of stent thrombosis at the 5-year follow-up were similar between DES and BMS groups (47). In this study, only one subject experienced asymptomatic stent occlusion, which was likely due to in-stent thrombosis caused by prolonged use of dual antiplatelet therapy. The 12-month rates of aspirin and clopidogrel usage were 84.4 and 76.0%, respectively. Future studies on the use of DESs for the treatment of VAS may provide an additional evidence that long-term dual antiplatelet therapy is essential.

There were several limitations to this study. Firstly, the lack of randomization precluded direct comparisons with optimal medical therapy or BMSs. As a single-arm trial, it was impossible to blind investigators, adjudicators, and personnel at the angiographic core laboratory. Secondly, to characterize a new implantable medical device such as the rapamycin-eluting stent, 6 months of angiographic follow-up and 12 months of clinical follow-up may be insufficient to observe all the occurrences of ISR, delayed stent thrombosis, and other late events. Thirdly, the small sample size limited our ability to perform additional analyses of whether certain patient subsets (especially those with V2 stenosis) have the lower ISR risk after placement of the rapamycin-eluting stents. Fourthly, we did not conduct a hemodynamic evaluation or acetazolamide challenge test before DES placement (48, 49). Finally, although a low dose of drug was released by the stent and there was no indication of rapamycin-induced neurotoxicity, further study is needed to assess the potential risk thereof in a neurovascular territory.

## Conclusion

The placement of the rapamycin-eluting stents in patients with symptomatic extracranial VAS yields favorable ISR results and showed a trend of favorable safety outcomes including low rates of perioperative complications and late stroke. However, further study is needed to establish the long-term clinical benefits of this stent in the treatment of the VA disease.

## Data Availability Statement

The raw data supporting the conclusions of this article will be made available by the authors, without undue reservation.

## Ethics Statement

The studies involving human participants were reviewed and approved by IRB of Beijing Tiantan Hospital, Capital Medical University. The patients/participants provided their written informed consent to participate in this study. Written informed consent was obtained from the individual(s) for the publication of any potentially identifiable images or data included in this article.

## Author Contributions

ZM and KC designed, led the study and had full access to all of the data in the study and takes responsibility for the integrity of the data and the accuracy of the data analysis. GM prepared the first draft of the report. WL did statistical analyses. All authors except WL participated in patient enrolment, collection of data. All authors critically reviewed the report and approved the final version.

## Funding

The authors declare that this study received funding from MicroPort NeuroTech (Shanghai, China). The funder was not involved in the study design, collection, analysis, interpretation of data, the writing of this article or the decision to submit it for publication. The authors designed the trial. Data analyses were performed by an independent institution (Department of Medicine Statistics, National Center for Cardiovascular Diseases, Beijing, China). KC and ZM had full access to all the data in the trial and assumes the final responsibility for the decision to submit the article for publication. All authors contributed to the article and approved the submitted version.

## Conflict of Interest

ZM has served as an expert consultant for MicroPort NeuroTech. WW and ZZ were the principal investigators of one completed and one ongoing clinical trial sponsored by MicroPort NeuroTech. JW was previously a principal investigator of a completed clinical trial sponsored by MicroPort NeuroTech. The remaining authors declare that the research was conducted in the absence of any commercial or financial relationships that could be construed as a potential conflict of interest.

## Publisher's Note

All claims expressed in this article are solely those of the authors and do not necessarily represent those of their affiliated organizations, or those of the publisher, the editors and the reviewers. Any product that may be evaluated in this article, or claim that may be made by its manufacturer, is not guaranteed or endorsed by the publisher.
