# Supplementary material for: Safety and Efficacy of Rapamycin-Eluting Vertebral Stents in Patients With Symptomatic Extracranial Vertebral Artery Stenosis
Source: Front Neurol. 2021 Nov 26;12:649426. doi: 10.3389/fneur.2021.649426 (PMC8662782; doi:10.3389/fneur.2021.649426)
Supplement: Supplementary file 1 [file Table_1.docx]

**Supplementary Appendix**

**Table S1.** **Severe adverse events within 12-month follow up.**

| **Event name** | **No. of SAEs** | **Patient ID** |
| --- | --- | --- |
| Angina | 3 | 01001, 01010, 03006 |
| Fracture of pubis | 1 | 02004 |
| Sudden deafness | 1 | 02005 |
| Renal arterial stenosis and stent placement | 1 | 02006 |
| Carotid artery stenting | 3 | 02008, 04407, 04410 |
| Lumbago | 1 | 02008 |
| Respiratory failure | 1 | 02010 |
| Pulmonary infection | 1 | 02010 |
| Sudden cardiac arrest | 1 | 02010 |
| Death | 3 | 02010, 06005, 06006 |
| Acute cholecystitis | 1 | 03003 |
| Anaemia | 1 | 03005 |
| Headache | 1 | 03006 |
| Anxiety neurosis | 2 | 03006, 03023 |
| Dizziness | 2 | 03009, 06004 |
| Lower limb numbness | 1 | 03017 |
| Pain | 1 | 03019 |
| Renal inadequacy | 1 | 03019 |
| Diabetic peripheral neuropathy | 2 | 03019, 06004 |
| Diarrhoea | 1 | 04402 |
| Arthralgia | 1 | 04403 |
| Thalamus hemorrhage | 1 | 04408 |
| Upper gastrointestinal bleeding | 1 | 04409 |
| Transient ischemic stroke (anterior circulation) | 1 | 06008 |
| Heart failure | 1 | 06010 |
| **Total** | **34** | **25** |
